# A Compact Optical Instrument with Artificial Neural Network for pH Determination

**DOI:** 10.3390/s120506746

**Published:** 2012-05-22

**Authors:** Sonia Capel-Cuevas, Nuria López-Ruiz, Antonio Martinez-Olmos, Manuel P. Cuéllar, Maria del Carmen Pegalajar, Alberto José Palma, Ignacio de Orbe-Payá, Luis Fermin Capitán-Vallvey

**Affiliations:** 1 Department of Analytical Chemistry, Campus Fuentenueva, Faculty of Sciences, University of Granada, E-18071 Granada, Spain; E-Mails: scapel@ugr.es (S.C.-C.); idorbe@ugr.es (I.O.-P.); 2 Department of Electronics and Computer Technology, Campus Fuentenueva, Faculty of Sciences, University of Granada, E-18071 Granada, Spain; E-Mails: nurilr@ugr.es (N.L.-R.); amartinez@ugr.es (A.M.-O.); ajpalma@ugr.es (A.J.P.); 3 Department of Computer Science and Artificial Intelligence, E.T.S. Ingenierías Informática y de Telecomunicación, University of Granada, E-18071 Granada, Spain; E-Mails: manupc@decsai.ugr.es (M.P.C.); mcarmen@decsai.ugr.es (M.C.P.)

**Keywords:** portable instrument, H coordinate, HSV colour space, pH sensor array, neural networks

## Abstract

The aim of this work was the determination of pH with a sensor array-based optical portable instrument. This sensor array consists of eleven membranes with selective colour changes at different pH intervals. The method for the pH calculation is based on the implementation of artificial neural networks that use the responses of the membranes to generate a final pH value. A multi-objective algorithm was used to select the minimum number of sensing elements required to achieve an accurate pH determination from the neural network, and also to minimise the network size. This helps to minimise instrument and array development costs and save on microprocessor energy consumption. A set of artificial neural networks that fulfils these requirements is proposed using different combinations of the membranes in the sensor array, and is evaluated in terms of accuracy and reliability. In the end, the network including the response of the eleven membranes in the sensor was selected for validation in the instrument prototype because of its high accuracy. The performance of the instrument was evaluated by measuring the pH of a large set of real samples, showing that high precision can be obtained in the full range.

## 1. Introduction

In general, optical pH sensors are based on reversible changes induced by pH in the structure of an acid-base indicator and translated into changes in spectroscopic phenomena such as absorption, reflectance, luminescence, and energy transfer. Nevertheless, some optical sensors are not based on acid—base indicators, such as those based on changes in the ionisation of unclad silica optic fibre by pH traced by methylene blue adsorption via evanescent field [1] and the pH-dependent polymer swelling of functionalised polymer microspheres dispersed in a hydrogel membrane that change the membrane turbidity [2].

According to the Henderson–Hasselbalch equation, the change in the measured signal with pH results is described by a narrow sigmoidal shape. Thus, the dynamic working range for pH optical sensors is limited to a few pH units (2–3) [3] and even shorter if the linear relationship in the middle of the sigmoidal response is used. This short range is one of the main drawbacks of these optical sensors for pH, along with their nonlinear response, which requires different sensing membranes to cover the whole pH range.

A common strategy to extend the working range of pH optical sensors consists of the preparation of arrays of sensing membranes containing complementary pH indicators that acquire the analytical information by imaging techniques. In this way, commercial multi-colour pH paper strips have been measured with a conventional scanner [4,5]; alternatively, arrays of five pH membranes in a triacetylcellulose support measured with a CCD colour camera have been described [6]. In both cases, the average RGB values of each sensing area image are used for calibration with multi-linear mathematical models. Suzuki *et al.* [7] developed a micro-well array chip system for parallel monitoring of single cell function, considering pH and oxygen concentrations as good indicators for cell activity. Fluorescein isothiocyanate (FITC) was used as a pH sensing indicator and the fluorescence intensity of each well was measured by using a high resolution microarray scanner.

One drawback of this calibration process is the non-linear dynamics of the functions to approximate the pH with respect to the colour parameters. Neural networks [8] have also been proposed to solve this problem, although the limitations of their classic training methods, becoming trapped in local optima solutions, may make the model calibration processes difficult [9]. Taib *et al.* [10] applied an artificial neural network for the analysis of the response of an optical fibre pH sensor (pH 2.51–9.76) based on a 3,4,5,6-tetrabromophenol sulphonephthalein indicator. The error produced during testing and application, using a network architecture of eight input neurons, 13 neurons in a single hidden layer and one output, was 0.08 and 0.07 pH, respectively. Other authors [11] have developed an optical pH sensor based on the immobilization of a mixture of two dyes on a triacetylcellulose membrane. The measuring range of the optode was extended by application of a back-propagation artificial neural network (ANN) to the whole pH range, providing mean square errors of 0.03 and 0.04 for test and control sets, respectively.

Previous work by our group has focused on the development of a disposable optical sensor array to predict the pH of a solution over the full-range (0–14). This pH determination was obtained from the hue (H) values of the HSV colour space using a scanner and custom developed portable instrumentation to image the optical array containing 11 sensing elements with immobilised pH indicators and using diverse mathematical modelling [12–14]. In the first case [12], three different approaches for pH prediction were studied: Linear models, Sigmoid competition models and Sigmoid surface models, providing mean square errors (MSE) of 0.111, 0.075 and 0.266, respectively, for tap and river water samples. Then, neural networks were used as a prediction technique [13]. The best network structure obtained with the traditional trial-and-error procedure and using the Levenberg–Marquardt training algorithm was made up of 11 input neurons, 10 hidden neurons and one output neuron for pH prediction, providing an MSE of 0.043. In the last case [14], a handheld instrument was presented for the measurement of pH using a disposable optical sensor array with more simple electronics than our previously designed photometers [15,16]. The acquisition of colour information from the array is obtained with a wide and programmable light source and a set of colour detectors that output the measured RGB coordinates coming from each membrane, in digital format, and used to calculate the H of the HSV colour space as the analytical parameter.

In this paper, the previously characterised portable instrumentation, which was optical and electrical, has been improved and the neural network programming optimised for the prediction of the pH in the full range. In our approach, the hue colour feature, obtained by means of imaging techniques from a sensor array, is used as the input for a neural network that provides the pH of a sample. This network is implemented in the microcontroller of the portable instrument. In addition, we also provide a procedure to minimise the number of sensing elements during calibration that could be used to achieve a suitable pH prediction in test and validation, with the aim of minimising the final instrument prototype cost and saving energy. We explored this problem previously [16] for scanned images in the laboratory, obtaining very accurate results. In this work, the hue colour parameter from each sensing element is extracted by the colour detectors implemented in the portable instrument to build the calibration data set. Then, a multi-objective algorithm provides a set of neural networks with maximum accuracy and the optimal sensing elements to be included in the sensor array.

This calculation method for pH prediction has been included in the memory of the microcontroller of the instrument. The prototype was then used to measure pH in a large variety of real samples, with good performance and little error.

## 2. Experimental

### 2.1. Reagents and Materials

The chemicals used for preparing the pH sensitive films were high molecular weight polyvinyl chloride (PVC), o-nitrophenyloctylether (NPOE), dioctyl sebacate (DOS), bis(1-butylpentyl)adipate (BBPA), tributyl phosphate (TBP), potassium tetrakis (4-chlorophenyl)borate (TCPB), tridodecyl-methylammonium chloride (TDMAC), Aliquat 336, cellulose acetate (CA), ethylene glycol and tetrahydrofuran (THF) all purchased from Sigma (Sigma-Aldrich Química S.A., Madrid, Spain). As acid-base indicators: 1-amino-4-hydroxyanthraquinone, sodium 3,3-[(1,1-biphenyl)-4,4-diyl(azo)] bis(4-aminonaphtalenesulfonate), N-[4-[bis[4-(dimethylamino)phenyl]methylene]-2,5-cyclohexadien-1-ylidene]-N-methylmethanaminium chloride (crystal violet), 2-(1,3-dihydro-3-oxo-2H-indol-2-ylidene)-1,2-dihydro-3H-indol-3-one (indigotin blue), 2-[2-[4-(dimethylamino)phenyl]diazenyl]-benzoic acid (methyl red), 4,4′-(1,1-dioxide-3H-2,1-benzoxathiol-3-ylidene)bis[2-bromo-3-methyl-6-(1-methylethyl)phenol (bromothymol blue), 4,4′-(1,1-dioxide-3H-2,1-benzoxathiol-3-ylidene)bis[2-bromo-6-methylphenol (bromcresol purple), N,N′-[(1,1-dioxide-3H-2,1-benzoxathiol-3-ylidene)bis[(6-hydroxy-5-methyl-3,1-phenylene)methylene]]bis[N-(carboxymethyl)-glycine (xylenol orange), 4,4′-(1,1-dioxide-3H-2,1-benzoxathiol-3-ylidene)bisphenol (phenol red), 4,4′-(1,1-dioxide-3H-2,1-benzoxa-thiol-3-ylidene)bis[5-methyl-2-(1-methylethyl)phenol] (thymol blue), 4,4′-(1,1-dioxide-3H-2,1-benzoxathiol-3-ylidene)bis[3-methylphenol] (m-cresol purple), N8,N8,3-trimethyl-2,8-phenazine-diamine hydrochloride (neutral red) and 1-phenylazo-2-naphthol (sudan I), 1-(2-pyridylazo)-2-naphthol (PAN) from Sigma, (1,2-benzo-7-(diethylamino)-3-(octadecanoylimino) phenoxazine (liphophilised Nile blue), 9-(diethylamino)-5H-benzo[a]phenoxazin-5-one (Nile red), 1,2,4-trihydroxy-9,10-anthracenedione (purpurin) and 4-(2-pyridylazo)resorcinol (PAR) from Fluka (Fluka, Madrid, Spain), 4,4′-(1,1-dioxide-3H-2,1-benzoxathiol-3-ylidene)bis[2-methylphenol] (cresol red) from Panreac (Panreac, Barcelona, Spain), 1,2-dihydroxy-9,10-anthracenedione (alizarine) from TCI (TCI Europe, Belgium), calcium 3-hydroxy-4-[2-(4-methyl-2-sulfophenyl)diazenyl]-2-naphthalene-carboxylate from BASF (BASF, Ludwigshafen, Germany), 1,4-dihydroxyanthraquinone and 1,5-dihydroxyanthraquinone from ICN (ICN K&K Laboratories, Plainview, NY, USA), 2,6-dihydroxy-anthraquinone from EGA (EGA Chemie, Steinheim, Germany) were used. Sheets of Mylar-type polyester (Goodfellow, Cambridge, UK) were used as support. HCl and NaOH were supplied by Sigma. All reagents were of analytical reagent grade and were used without any further purification. All solutions were prepared in reverse-osmosis type quality water (Milli-RO 12 plus Milli-Q station from Millipore, conductivity 18.2 Mohm/cm).

### 2.2. Instruments and Software

The optical spectra were measured with a mini-spectrometer RC series C11007MA (Hamamatsu Photonics, Hamamatsu, Japan) with 256 pixels, spectral resolution at 9 nm half width and 16 bits of intensity resolution. For the electrical characterisation of the prototype, the following instrumentation was used: a mixed signal oscilloscope (MSO4101, Tektronix, Beaverton, OR, USA), a 6½ digit multimeter (34410A, Agilent Technologies, Palo Alto, CA, USA), a 15 MHz waveform generator (33120A, Agilent Technologies) and a DC power supply (E3630A, Agilent Technologies). For the image acquisitions and digitalisation, a commercial scanner ScanMaker i900 (Microtek, Hsinchu, Taiwan) was used, with a 6,400 × 3,200 dpi resolution, a maximum optical density of 4.2 and 24 to 48 bits of colour. The software to manage the scanner was Silver Fast Ai provided by Microteck. The images were processed with a set of scripts and functions developed by us in Matlab r2007b (The MathWorks, Inc., Natick, MA, USA). Absorbance measurements of the membranes for comparative purposes were performed by a Hewlett Packard diode array spectrophotometer (model 8453; Nortwalk, CT, USA) equipped with a homemade membrane cell holder. Statistical calculations were performed with the Statgraphics software package (Manugistics Inc. and Statistical Graphics Corporation, Rockville, MD, USA, 1992), and Microsoft Excel (Microsoft Corp., Redmond, WA, USA) was used for general calculations. A Crison model Basic 20 pH-meter (Crison Instruments, Barcelona, Spain,) with a combined double junction glass electrode, calibrated against two standard buffer solutions (pH 4.0 and 7.0), was used for the pH measurements.

### 2.3. pH Sensor Array Preparation

The sensor array was prepared on a 5 cm × 4 cm transparent Mylar polyester support covered with an adhesive black film of PVC with 12 holes (3 columns and 4 rows), 5 mm in diameter each. A black opaque film was used to reduce the light dispersion and prevent cross information between the sensing elements, and one empty position of the support (S4) was used to test the illuminant of the instrument. The sensing films were cast by carefully placing 8 μL of the corresponding cocktail in each hole, whose surface tension and quick evaporation make it possible to prepare the sensing membrane. The different cocktails for the pH membranes were prepared by dissolving the different chemicals needed in 1 mL of distilled THF according to the following composition. 1: 1.00 mg of sicomet red P, 4.00 mg of Aliquat 336, 47.00 mg of NPOE and 19.00 mg of PVC; 2: 2.10 mg of *m*-cresol purple, 9.40 mg of TDMAC, 19.60 mg of DOS, 23.10 mg of CA and 15.80 mg of ethylene glycol; 3: 1.00 mg of PAN, 7.00 mg of TDMAC, 45.50 mg of NPOE and 16.52 mg of PVC; 4: 0.50 mg of purpurin, 2.90 mg of TDMAC, 23.45 mg of NPOE and 8.15 mg of PVC; 5: 0.50 mg of cresol red, 1.59 mg of Aliquat 336, 23.45 mg of NPOE and 9.46 mg of PVC; 6: 0.50 mg of liphophilised Nile blue, 1.27 mg of TCPB, 23.45 mg of TBP and 9.77 mg of PVC; 7: 2.10 mg of bromothymol blue, 2.80 mg of TDMAC, 19.60 mg of DOS, 25.90 mg of CA and 19.60 mg of ethylene glycol; 8: 0.50 mg of alizarin, 3.60 mg of TDMAC, 23.45 mg of NPOE and 7.45 mg of PVC; 9: 1.10 mg of thymol blue, 4.05 mg of TDMAC, 9.80 mg of DOS, 12.95 mg of CA and 7.15 mg of ethylene glycol; 10: 1.00 mg of phenol red, 4.85 mg of TDMAC, 18.20 mg of BBPA, 21.00 mg of CA and 24.95 mg of ethylene glycol; 11: 3.50 mg of thymol blue, 12.88 mg of TDMAC, 19.60 mg of DOS, 23.10 mg of CA and 10.92 mg of ethylene glycol.

The pH sensing elements containing colorimetric acid-base indicators were selected according to the conditions of: (a) no leaching; (b) a tonal colour coordinate change by reaction; and (c) full coverage of the pH range by overlapping the responses of the different membranes. The selected sensing elements were prepared from different cocktails containing different types and amounts of colorimetric acid-base indicators, polymers, plasticisers, lipophilic salts and, if necessary, humectant.

The selection criteria for the pH membranes used were both high variation in H coordinate by reaction and non-redundant information from different sensing elements, so that the entire pH range was covered. At times, to displace the pH response of a membrane, we used the same acid-base indicator but changed the plasticiser, membrane polymer, lipophilic salt and/or lipophilic salt/indicator ratio. As a result, 11 different membranes, containing 10 different pH indicators were prepared to cover the whole pH range. The composition of the different sensing elements was optimised considering leaching minimisation (lipophilic salt, plasticiser, and membrane polymer), colour intensity (acid-base indicator) and response time (plasticiser, membrane polymer, humectant and cocktail volume).

### 2.4. Description of the Instrument

**Figure 1.**
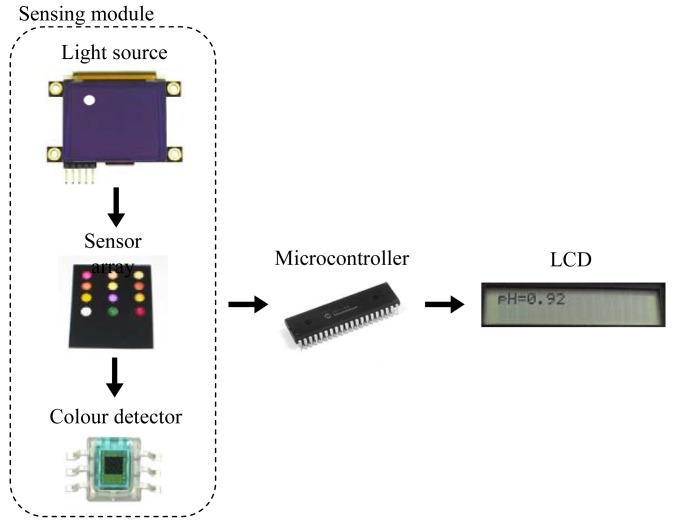
Instrument sensing design.

The portable instrument used in this work is a microcontroller-based system designed to measure the pH of a solution using a neural network. The neural network implemented relates the H component from the HSV colour space of a set of colorimetric sensing elements to the pH. A diagram of the sensing module in the instrument is presented in Figure 1. An organic light-emitting diode (OLED) display works as light source by illuminating the sensing elements of the array. The wavelengths transmitted by the sensor array being illuminated are captured by digital colour detectors which provide RGB measurement to the microcontroller. This device obtains the corresponding H coordinates in order to determine the pH value. A keypad and a Liquid Crystal Display (LCD) screen allow the user to interact with the prototype, working as user interface.

Due to the different modules present in the design and the resources needed by the neural network, a PIC18F4550 model (Microchip Technology Inc., Chandler, AZ, USA) was selected since it has five input-output ports, 24 KB of flash memory and 2 KB of SRAM. The OLED display used as light source, model 160-GMD1 (4DSystems, Sydney, Australia), illuminates each element of the sensor array in a sequential way by displaying the circles with the illuminant for each position, as shown in Figure 1. The main reason for using a display as the illuminant is the possibility of programming different configurations for the RGB components in order to obtain an illuminant similar to D65, which is widely used in colorimetric applications.

The transmitted wavelengths by the illuminated sensing elements are captured with a set of S9706 digital colour detectors (Hamamatsu Photonics, Hamamatsu City, Japan) which provide RGB information by means of a 36-bit word output which are serially sent to the microcontroller for further processing. The main configuration parameter of the detectors is the integration time that determines the interval during the photodiode matrix generates a photocurrent. A trade-off between sensitivity and response time determines this parameter and, in this case, a 200 ms time was programmed. The total measurement time is about 2.5 seconds, including the acquisition and processing times.

**Figure 2.**
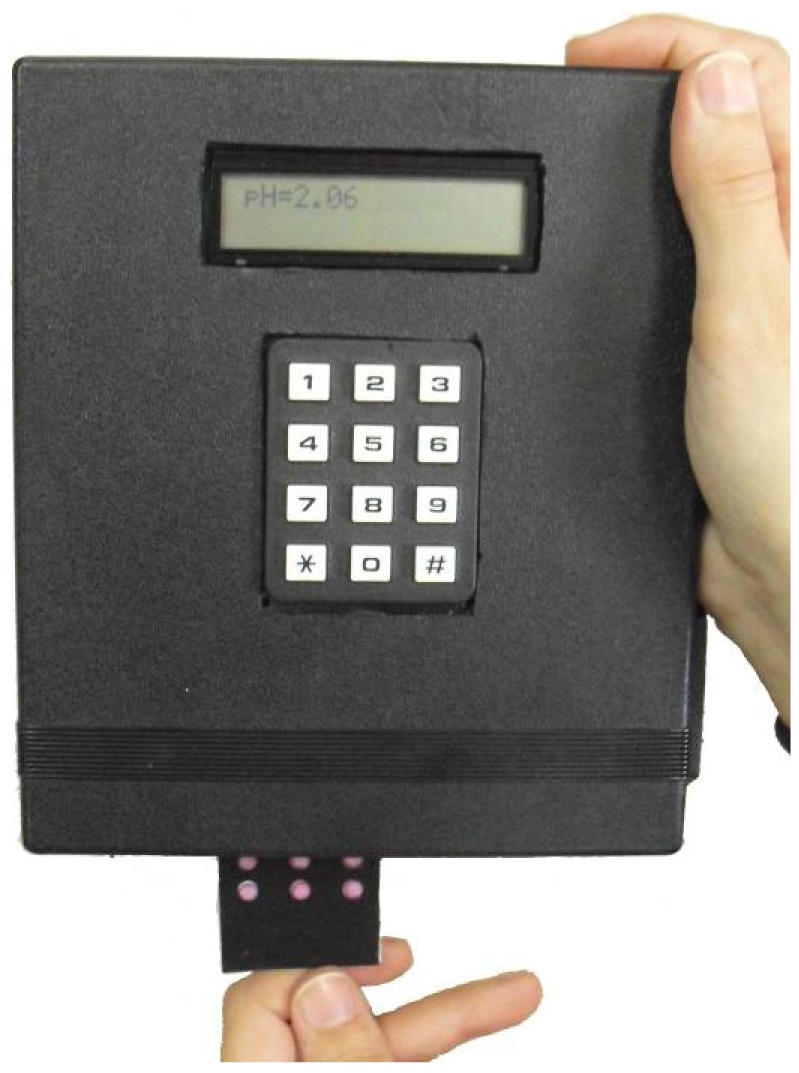
Photograph of the prototype.

In Figure 2, a photograph of the described prototype is presented. The keypad allows the user to change different parameters like integration time, illuminant or measurement mode for calibration purposes. An LCD is included in the design to show the different selectable options and the results of the pH obtained for each measurement. At the bottom of the instrument there is a slot to insert the sensor array. The prototype is integrated in a closed box to avoid interference with the ambient light. The device power consumption has been evaluated in two situations: while measuring, it is 450 mW; in a stand-by mode, the consumption is 5 mW.

### 2.5. pH Determination Model

Different approaches were considered in previous works as pH determination models. Imaging techniques and multivariate sensor response modelling developed in [12] have high computer memory and processor speed requirements, which are important limitations for the implementation on portable devices. On the other hand, neural networks were tested to overcome these problems in [13], obtaining higher accuracy with lower requirements in computer resources. In addition, neural networks and specifically Perceptron and recurrent models can be optimized and minimized using multi-objective optimization techniques [16], which allow us to reduce the number of sensing elements in the sensor array. For this, reason, in this work neural networks are used as prediction models for pH determination from colour features of the sensing elements in the array. A Multilayer Perceptron [8] with one hidden layer was designed for this purpose, where the hue (H) colour component from each sensing element is a candidate input for the network, and the output is the predicted pH value. The activation function for all hidden neurons was set to the sigmoid function, and the output neuron response is computed as the weighted linear aggregation of the output value provided by intermediate hidden neurons. Considering our aims of saving energy, minimising the array and final instrument prototype, and resource management, we were also interested in discovering the minimum number of sensing elements in the array needed to provide a fine pH prediction. To achieve this, the neural network to be implemented in the instrument prototype was obtained using a multi-objective procedure with three criteria to be minimised (Equation (1)): (a) criterion f_1_(x) (Equation (2)) to minimise the number of sensing elements used for pH prediction, (b) criterion f_2_(x) (Equation (3)) to minimise the network prediction error, and (c) criterion f_3_(x) (Equation (4)) to minimise the number of hidden neurons. The first and second objectives attempt to obtain the sensing elements required to provide accurate pH approximation with minimum error. The error measure to be minimised is the maximum absolute error between the network response and all the calibration data. This error measurement does not depend on the number of training data, and is suitable to prevent overtraining when the data are unbalanced [16], *i.e.*, some pH ranges are better approximated than others. Finally, the third objective is used to reduce the microchip memory and processing consumption, and it also helps to avoid overtraining of networks with an excessive number of hidden neurons. These criteria are formulated in Equations (1) to (4), where *x* stands for the neural network parameters to be optimised, x* is the set of optimal solutions, *pH_p_* is the real pH measured for the *p*-th sample in the calibration data, *pH′_p_* is the pH approximation provided by the neural network, and *N^0^*(*x*) and *N^1^*(*x*) are the number of network neurons at input and hidden layers, respectively:

(1) $$x^{*}=\min_{x}\{(f_{1}(x),f_{2}(x),f_{3}(x)\}$$

(2) $$f_{1}(x)=N^{0}(x)$$

(3) $$f_{2}(x)=\max_{p}\left\{\left|{pH^{\prime }}_{p}-pH_{p}\right|\right\}\forall p$$

(4) $$f_{3}(x)=N^{1}(x)$$

A hybrid version of the NSGA-II multi-objective algorithm has been designed in combination with the Levenberg-Marquardt non-linear optimisation method using the Baldwinian hybridation strategy [16]. The resulting method is able to provide a set of optimal neural networks according to the Pareto optimality criterion. In brief, the objective is to find a neural network or set of neural networks with optimal (minimum) values in all criteria [f_1_(x), f_2_(x), f_3_(x)]. To achieve this, the *dominance* over solutions of the multi-objective algorithm is defined in equation 5 In this work, a neural network *x* dominates the neural network *y*, and it reads *x* < *y*, if the solution *y* is not better than *x* in any of the criteria to be optimised, and also *x* is better than *y* in at least one of the three criteria. If a solution *x* does not dominate *y* and also *y* does not dominate *x*, both are *non-dominated solutions*. The *Pareto front* is composed of the set of solutions that are not dominated by any other solution. For the purpose of this work, the developed method in [16] is able to provide a Pareto front containing a set of networks that are optimal under this optimality criterion.

(5) $$x<y↔\forall i\in \{1,2,\ldots ,n\}f_{i}(x)\leq f_{i}(y)\wedge \exists j\in \{1,2,\ldots ,n\}:f_{j}(x)<f_{j}(y)$$

**Figure 3.**
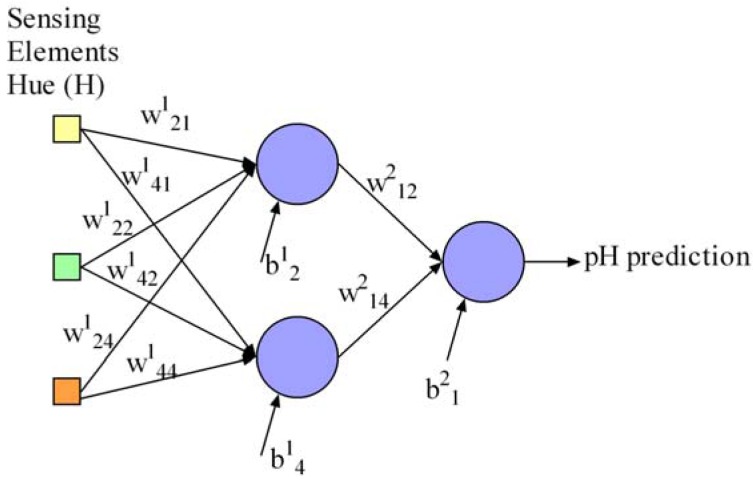
Example of the neural network described in Equations (6–9).

Additionally, we define the neural network parameters x to be optimised in this work regarding the pursued objectives. It is necessary to optimise the network weights and biases to achieve a suitable pH determination, the number of sensing elements in the array for minimisation of array and instrument prototype development cost and size, and the number of hidden neurons for processor energy consumption savings, processing speed and overtraining prevention. These parameters are represented in the proposed computer optimisation algorithm as follows: (a) a matrix *W* containing the network connection weights as real numbers, (b) a vector *B* with the bias values of the neurons as real numbers, (c) a vector *I* with binary values to indicate which sensing elements in the array are used for pH determination in the network, and (d) a vector *H* with binary values to indicate the network hidden neurons. This neural network representation is supported by a widely-used proposal in the literature for the multi-objective optimisation of neural networks [17], and has the advantage of being flexible enough to be used for the optimisation of more complex neural network models such as recurrent neural networks. Our contribution to this representation is the possibility of optimising the number of network inputs and was validated in [16]. For the sake of clarity, Equations (6) to (9) describe a possible representation of a neural network with a maximum of five inputs (Equation (8)) and four hidden neurons (Equation (9)) as an example. Inputs 1, 2, 4 are active and would represent the sensing elements used in the array for pH determination in our work. The network contains two hidden neurons called 2 and 4. The network weights and biases are represented in Equations (6) and (7), respectively. Figure 3 draws the resulting network from this example:

(6) $$W=\left(\begin{array}{ccccccccc} w_{1,1}^{1} & w_{1,2}^{1} & w_{1,3}^{1} & w_{1,4}^{1} & w_{1,5}^{1} & 0 & 0 & 0 & 0\\ w_{2,1}^{1} & w_{2,2}^{1} & w_{2,3}^{1} & w_{2,4}^{1} & w_{2,5}^{1} & 0 & 0 & 0 & 0\\ w_{3,1}^{1} & w_{3,2}^{1} & w_{3,3}^{1} & w_{3,4}^{1} & w_{3,5}^{1} & 0 & 0 & 0 & 0\\ w_{4,1}^{1} & w_{4,2}^{1} & w_{4,3}^{1} & w_{4,4}^{1} & w_{4,5}^{1} & 0 & 0 & 0 & 0\\ 0 & 0 & 0 & 0 & 0 & w_{1.1}^{2} & w_{1.2}^{2} & w_{1.3}^{2} & w_{1.4}^{2} \end{array}\right)$$

(7) $$B=(\begin{array}{ccccc} b_{1}^{1} & b_{2}^{1} & b_{3}^{1} & b_{4}^{1} & b_{1}^{2} \end{array})$$

(8) $$I=(\begin{array}{ccccc} 1 & 1 & 0 & 1 & 0 \end{array})$$

(9) $$H=(\begin{array}{cccc} 0 & 1 & 0 & 1 \end{array})$$

### 2.6. Measurement Procedure

The colour determination of the pH membranes was performed by measuring the transmitted light following the path indicated in Figure 1: once the sensor array board is inserted into the instrument (see Figure 2), the light is generated in the programmable OLED that acts as a white light source and illuminates the colour sensors in the array. This illumination is carried out in a sequential way by placing a white circle drawn on a black background facing the corresponding colour sensor. Each sensing element of the array works as a light filter and only transmits the wavelengths corresponding to its own colour. This transmitted light reaches the surface of the colour detectors and they generate a digital output with the values of the RGB decomposition of the incident light, which is used to characterise the colour of the sensing elements.

The response of the measurement system was evaluated for each 0.1–0.2 pH unit from 0 to 14 by adding volumes of 1.0 M, 0.1 M or 0.01 M of HCl or of NaOH with a microburette to an aqueous solution containing the sensor array hanging from a support with a clamp. After each addition and magnetic stirring, the pH of the solution was measured using a potentiometric procedure. The array sensor was equilibrated for 5 min, and then was pulled out and inserted into the instrument to be measured. All measurements were made in an air-conditioned laboratory at room temperature (24 ± 1 °C).

## 3. Results and Discussion

The RGB space is an additive representation of colour in which all the colours can be represented as a combination of red, green and blue primaries. The colour space used here, HSV, is an alternate representation of colour derived from the red, green, and blue intensity values of the RGB space. A pixel in this colour space is defined by its hue (H), saturation (S), and value (V) coordinates. In broad terms, H is a numerical representation of the colour, S gives the degree to which a single channel dominates, and V represents the brightness. We have demonstrated previously that the H value is stable, simple to calculate, and easily obtained from commercial devices, maintaining a superior precision with variations in indicator concentration, membrane thickness, detector spectral responsivity, and illumination [18]. Thus, the main reason to use H, which only considers hue variations of sensing elements and not intensity variations connected to S and V coordinates, is to prevent problems such as dye leaching or lot-to-lot variations. The evolution of S and V components with pH for different membranes was studied, observing small variations except in some cases, and in general they do not improve the results. Furthermore, the use of the S and V parameters involves a more complex treatment and a high consumption of computational time.

**Figure 4.**
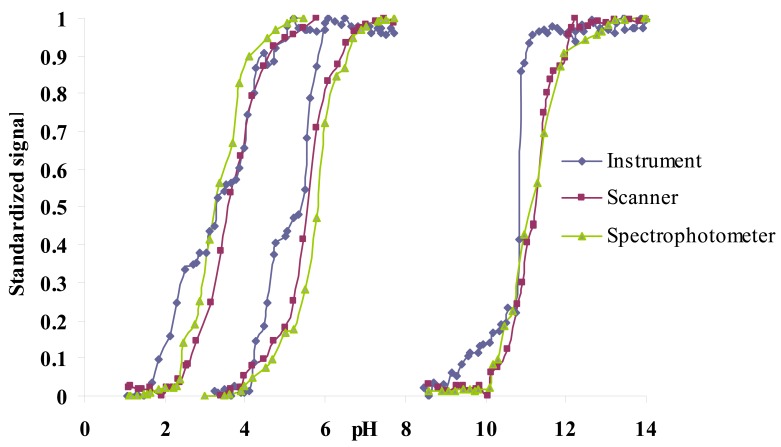
Comparison between the responses to pH of different sensing elements using different devices for three array sensors.

In a previous work, the same sensor array was individually characterised by means of spectrophotometry and scannometry, where imaging techniques were used to acquire the tonal information (H coordinate) of the HSV colour space [9]. As a first step, the H values obtained with our portable instrument were compared to the aforementioned laboratory techniques. The results obtained with the portable instrument are very similar to those obtained using imaging techniques to acquire the tonal information or absorbance measurements as shown in Figure 4 for three colorimetric sensing elements of the array.

### 3.1. Calibration and Neural Network Selection

The experiment was designed so that the pH range from 0 to 14 was uniformly sampled with 121 solutions to build the calibration data set. A sensor array was immersed in each solution and we made 12 replicates to prevent any outliers. Then, the hue (H) value of the 11 sensing elements in the sampled arrays was measured with the portable instrument and saved in the computer for neural network calibration. The multi-objective hybrid NSGA-II algorithm [16] was executed 30 times with these calibration data as input for better exploration of solution space and Pareto front acquisition, using the following parameters:

- Bounds for the number of hidden neurons: 2 (minimum) and 11 (maximum).
- Bounds for the number of sensing membranes considered: 1 (minimum) and 11 (maximum).
- Bounds for network biases and weights: [−10, 10].
- Number of layers of the neural networks: 1 (input), 1 (hidden), 1 (output).
- Crossover probability: 0.7.
- Mutation probability: 0.2 (number of sensing membranes used), 0.2 (number of hidden neurons), 0.2 (network weights), 0.1 (mutation per gene).
- Population size: 100.
- Number of algorithm generations: 700.
- Number of local search iterations with Levenberg-Marquardt: 10.

The hybrid NSGA-II procedure returned 21 non-dominated solutions in the Pareto frontier among the 30 executions, meeting the three objectives: number of sensing elements, hidden neurons, and pH prediction error minimisation. The performance of these solutions is summarised in Table 1, which also includes the Mean Square Error (MSE) obtained in the calibration data set for comparison purposes with other techniques in the literature. For reading clarity, we describe in depth the sensing elements of the array used for pH prediction by each resulting network in column 5. We remark that the maximum absolute error of the networks obtained is high in the calibration data, but this is justified because all the replicates of the measurements in this data set were used for neural network training. We made the decision to improve the network noise tolerance that could be produced during the physical hue acquisition in the portable instrument, and such measurements could be interpreted as outliers. Nevertheless, the error in validation and testing is lower because we removed the outliers.

As shown in Table 1, the solutions obtained cover a wide set of neural networks ranging from networks with low complexity, a low number of sensing elements and low accuracy to networks with high accuracy but also higher requirements of sensing elements necessary for the pH prediction. Some relevant results are networks 1, 10, 12, and 18. In the case of network 1, this network is able to obtain an MSE of 0.048 using seven sensing elements and a very low complexity of four hidden neurons. Networks 10 and 12 make use of all the sensing elements in the array and provide a similar MSE of 0.031 and 0.028, respectively, but network 10 has a lower complexity of six neurons in contrast to network 12, which has a 9-neuron complexity. Finally, network 18 is able to obtain an MSE of 0.065 using four sensing elements only, and a medium complexity of five neurons. The main advantage of this multi-objective procedure used to calibrate the pH prediction method is that all the networks provided in Table 1 are optimal under the Pareto optimality criterion. Thus, the designer of the final portable instrument prototype could choose from a wide set of solutions to be implemented in the instrument and s/he could decide which to use, using different selection criteria such as instrument size, array development cost, computing time and accuracy.

To validate the portable instrument developed in this work, we selected network number 12 in the Pareto front to be included in the hardware, since it provides both the lowest MSE and maximum absolute error in pH determination. This selection is discussed in Table 1. The network was implemented in C language and compiled using the PIC programming toolkit from Microchip for validation and testing. The code needed to implement this neural network in the program of the microcontroller was optimised to require only 10% of the available microcontroller's program memory. Therefore, the microcontroller selected here was able to perform all the signal processing necessary to generate the pH value prediction with no need for additional resources such as external memories.

### 3.2. Validation and Test

A set of 50 equally spaced solutions covering the full pH range was used to validate the portable instrument, making measurements with five replicates of the array sensor in each case. In order to test the prediction performance of the method, we applied a Student's t-test with a confidence level of 95% to check whether the data distributions resulting from the predicted values in the validation data set differ significantly from the real data (using a potentiometric method as reference). The probability value obtained was 0.984 (higher than 0.05), so we may conclude that the model is able to predict pH suitably since there are no significant differences between the real and predicted pH data. We also applied a Pearson correlation test to measure the quality of the predicted versus the real values. The result of the test provided a probability value under 2.2 × 10^−16^ in the validation data set, and we may therefore conclude that there is a significant correlation between the real and predicted values. The correlation coefficient R^2^ was calculated and the test provided the value 0.999. Consequently, it may be assumed that the prediction model provides a suitable performance for the task of pH prediction in the validation data. The pH predictions fit the original pH values with high fidelity in the range from 0 to 14, although the accuracy decreases for pH values higher than 13, due to the lower H variation of the sensor array in that range. The MSE obtained in the validation data is 0.014 and the maximum absolute error in pH units is 0.296, which is produced in the pH range 13–14, as described previously. To finish the analysis of the portable instrument validation, we separated the validation data into two sets to distinguish the acid and basic pH range. The samples in acid range 0–7 contained 27 measurements with five replicates for each one, and the samples in basic range 7–14 contained the remaining 23, also with five replicates for each sample. We calculated the absolute error between the pH prediction and the reference pH for each sample, and compared the error data distributions with a Student's t-test with 95% of confidence level, to discover whether the pH prediction is uniform across the full pH range or if the portable instrument performs better in a specific pH range. We obtained a probability value of 0.227 (over 0.05), so that we may conclude that there are no significant differences in the error committed by the portable instrument either in acid or basic pH ranges, and the method provides a uniform error in the full pH range.

Additionally, the procedure was applied to 10 aqueous solutions to test the prediction model (test set), with five replicates again, with the pH adjusted with acid and base, sampling the full pH range from 0 to 14 uniformly. The MSE obtained in this case is 0.015, and the maximum absolute error in pH units is 0.229. Compared to laboratory experiments carried out in previous works [16], the results for pH determination are similar using the device developed in this work, which suggests that the proposed portable instrument and the methodology followed can be used for pH prediction. Again, we applied a Student's t-test with a confidence level of 95% to test whether the pH prediction differs significantly from the pH measured with the reference method. The probability value obtained was 0.997, which indicates that there are no significant differences between pH prediction and reference pH data. The Pearson correlation test was applied to check if the predicted data match the real pH measurements, obtaining a probability value under 2.2 × 10^−16^. Thus, this test concludes that there is a significant correlation between the real and predicted values, with a correlation coefficient R^2^ of 0.999.

**Figure 5.**
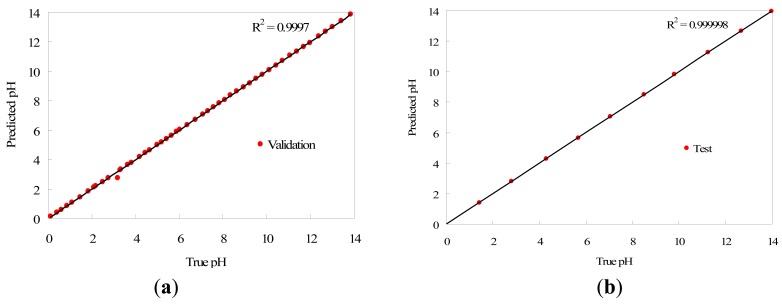
(**a**) Validation and (**b**) test measurements.

The correlation graph of pH predicted values against reference pH data for both validation and test data sets is presented in Figure 5, where they have been fitted to straight lines that correspond to the reference pH. Measured and reference values of pH match with high accuracy, as it can be deduced from the correlation factor R^2^ that is higher than 0.999 in both cases.

### 3.3. Application to Real Samples

The pH of beverages, personal care products and cleaning samples such as orange, tangerine, tomato, peach, grape and pineapple juices; orange, lemon and cola soft drinks; milk; tap, river, mineral and carbonated waters; vinegar; lemon; alcohol-free and draught beers; ammonia solution; washing-up liquid; toothpaste solution; mouthwash, *etc.* was determined, validating the results against a pH-meter (Table 2).

These products were used to validate the pH prediction capability of our portable instrument. An array was introduced into these products to measure their pH, and we used a pH-meter to validate the portable instrument response. In addition, we scanned the arrays and applied the method developed in [16] to calculate the pH using imaging techniques. We compared the results of pH prediction using both techniques (portable instrument and imaging) to validate the electronic performance of the proposed device in contrast to previous work. Table 2 shows the results obtained with the pH-meter and both imaging and portable instrument techniques for all samples described in column 1. Column 2 describes the pH of the solution measured with the pH-meter, columns 3–4 show the average pH prediction and absolute error regarding the pH-meter with imaging techniques, columns 5–6 the average pH prediction and absolute error with our portable instrument. In almost all cases, the pH prediction using the portable instrument is an improvement over the imaging procedure using the scanner. We found only one sample in which we obtained an absolute error over 1 pH unity, the bleach solution (1:5). This occurred because the same sensing elements in the array react with the solution and become spoiled, and the hues measured by both scanner and portable instrument do not fit the real solution pH.

To validate our assumption, a statistical t-test with a 95% confidence level was applied to the error distributions in the overall results. The data from the bleach solution (1:5) were removed to prevent any outliers. A probability value of 0.006 was obtained, meaning that there are significant differences between the results from the scanner imaging and portable instrument. In this case, the portable instrument provides better results than scanner imaging since its average error is 0.09 and the average error of scanner imaging is 0.13, as shown in Table 2. In addition, we studied the performance of the portable instrument for each solution class separately. We applied a means test with a 95% confidence level to compare the error distributions of pH prediction between scanner imaging and the portable instrument for the 40 samples separately. Column 7 in Table 2 describes the resulting probability value for each case. We have highlighted those rows to distinguish the classes where we found significant differences in pH prediction. The pH prediction results in 31 of 40 samples have no statistical difference, whether we use scanner imaging techniques or our portable instrument. Regarding the remaining solutions, the portable instrument provided better results in 6 cases of the remaining 9 samples. These analyses show that the portable instrument is able to predict the pH of real samples accurately and at times it can provide even better results than other non-portable pH prediction techniques.

## 4. Conclusions

In this work, we have proposed a programmable portable instrument for full range pH determination using colorimetric acid-base indicators. A prototype of the device was developed that can acquire the hue (H) component of the HSV colour space from an array containing a maximum number of up to 12 sensing elements. An array with 11 sensing elements was designed in order to obtain colorimetric responses in the full pH range from 0 to 14. The response of some of these sensing elements overlaps, making it possible to calibrate an accurate pH prediction model. Regarding the calibration methodology, neural networks were used as the pH prediction technique, and we designed a multi-objective neural network calibration procedure with the aim of minimising prediction model complexity, maximising accuracy and minimising the number of sensing elements. The method returns a set of optimal solutions, in the sense of Pareto optimality, to provide the designer of the final instrument prototype with a large set of solutions to be implemented in the portable instrument. Thus, the designer can implement an optimal solution considering further industrial development decisions such as energy saving, computing time response, array development costs and instrument minimisation, among others. The portable instrument was validated with a neural network that uses an input array with 11 sensing elements and provides the best accuracy, and we obtained a Mean Square Error of 0.028 in calibration data, 0.014 in validation data, and 0.015 in test data. In addition, a maximum absolute error of 0.229 in pH units was obtained with the test data. Statistical tests concluded that our method does not provide pH prediction that differs significantly from pH measurements using potentiometric techniques. Finally, we applied our method to test pH prediction capabilities in real samples such as orange juice, vinegar, cola drink, lemon dressing, tea and beer, among others. The results obtained are promising, since most of samples obtained an absolute error of 10^−2^ order units in comparison with the potentiometric method. In addition, we compared these results with previously developed imaging techniques in the literature using a scanner and computer software to make the pH prediction. Statistical tests concluded that there are no significant differences between the method using imaging techniques and our portable instrumentation. Thus, the portable device developed has been validated with previous works and it has been shown that it is able to provide a fine and accurate pH prediction.

## Figures and Tables

**Table 1.** Performance of resulting networks from hybrid NSGA-II in calibration.

**Solution Number**	**Maximum Absolute Error (pH units)**	**Number of Hidden Neurons**	**Number of Network Inputs**	**Sensing Elements Used for pH Prediction**	**MSE (Calibration Data)**
1	0.873	4	7	3 4 5 6 8 9 11	0.048
2	1.098	3	3	4 5 10	0.114
3	0.864	5	7	3 4 5 6 8 9 11	0.048
4	1.656	2	2	7 9	0.508
5	1.014	3	4	4 5 10 11	0.080
6	1.279	2	3	4 5 10	0.141
7	1.079	2	5	1 4 5 10 11	0.113
8	1.099	2	4	4 5 10 11	0.118
9	0.910	8	6	4 5 6 8 9 11	0.047
10	0.766	6	11	1 2 3 4 5 6 7 8 9 10 11	0.031
11	0.883	3	7	3 4 5 6 8 9 11	0.057
**12**	**0.734**	**9**	**11**	**1 2 3 4 5 6 7 8 9 10 11**	**0.028**
13	0.917	5	5	1 4 5 8 9	0.064
14	0.959	3	6	1 4 5 6 9 11	0.071
15	0.757	7	9	1 2 3 4 5 6 7 9 11	0.037
16	1.428	3	2	5 7	0.116
17	3.058	2	1	7	1.026
18	0.978	5	4	4 5 9 10	0.065
19	0.980	3	5	3 4 5 9 10	0.073
20	0.924	4	6	1 2 4 5 9 10	0.061
21	0.945	4	5	1 4 5 9 10	0.066

**Table 2.** Comparison between the pH of different samples measured with different devices.

**Sample**	**Potentiometer**	**Scanner**	**Error**	**Portable Instrument**	**Error**	**P-value**
**Lemon dressing**	**2.35**	**2.19**	**0.16**	**2.31**	**0.04**	**0.016**
**Tonic**	**2.49**	**2.59**	**0.10**	**2.39**	**0.10**	**0.027**
**Orange soft drink**	**2.75**	**2.83**	**0.08**	**2.67**	**0.08**	**0.044**
**Orange fizzy drink**	**2.91**	**2.98**	**0.07**	**2.92**	**0.01**	**0.035**
Blackberry liqueur	2.91	3.08	0.17	2.95	0.04	0.107
**Lemon fizzy drink**	**3.30**	**3.43**	**0.13**	**3.34**	**0.04**	**0.021**
Cola fizzy drink 1	2.85	2.78	0.07	2.89	0.04	0.055
**Cola fizzy drink 2**	**2.75**	**2.84**	**0.09**	**2.71**	**0.04**	**0.024**
Vinegar	3.03	2.99	0.05	3.09	0.06	0.616
White wine	3.35	3.29	0.05	3.36	0.02	0.117
Mandarin juice	3.57	2.44	0.13	3.52	0.04	0.228
Pineapple juice	3.68	2.69	0.01	3.67	0.02	0.633
Tomato juice	4.17	4.23	0.06	4.11	0.05	0.354
Orange juice	3.49	3.45	0.04	3.56	0.07	0.869
Apple juice	3.20	3.10	0.10	3.16	0.04	0.191
Peach juice	4.01	4.12	0.12	4.06	0.05	0.208
Grape juice	3.78	3.70	0.08	3.68	0.10	0.660
**Tinned gherkins**	**2.89**	**2.72**	**0.17**	**2.91**	**0.02**	**0.033**
**Tinned artichokes**	**3.80**	**3.78**	**0.02**	**3.72**	**0.08**	**0.021**
Tinned mushrooms	4.96	4.86	0.10	5.02	0.06	0.296
Tinned soya	3.97	4.07	0.10	4.05	0.08	0.558
Tinned olives	4.07	4.14	0.07	4.09	0.02	0.183
Tinned carrots	3.76	3.72	0.04	3.78	0.02	0.265
Beer	4.16	4.30	0.14	4.24	0.08	0.176
Alcohol-free beer	4.50	4.25	0.25	4.38	0.12	0.116
Black tea	4.93	5.08	0.15	5.21	0.28	0.068
Decaffeinated coffee solution	5.67	5.33	0.34	5.42	0.25	0.311
Skimmed milk	6.70	6.59	0.11	6.61	0.09	0.822
Sparkling mineral water	5.30	5.29	0.01	5.36	0.06	0.292
Mineral water 1	7.17	7.19	0.02	7.14	0.03	0.149
Mineral water 2	7.29	7.66	0.36	7.35	0.06	0.217
Mineral water 3	7.59	7.58	0.01	7.62	0.03	0.892
Tap water	7.85	7.97	0.12	7.96	0.11	0.294
Toothpaste solution (1:3)	7.22	7.31	0.09	7.36	0.14	0.297
Mouthwash	7.30	7.53	0.23	7.43	0.13	0.178
Diluted washing-up liquid	7.30	6.87	0.42	7.02	0.28	0.299
Contact lens saline solution	7.54	7.35	0.20	7.40	0.14	0.442
**Bleach solution (1:5)**	**8.99**	**2.52**	**6.47**	**3.42**	**5.57**	**0.030**
Sodium bicarbonate saturated solution	8.44	8.37	0.07	8.30	0.14	0.527
Ammonia solution (1:10)	11.04	11.46	0.42	11.58	0.54	0.312
		Average error:	0.13	Average error:	0.09	
